# Assessing Return to Performance After Surgery in National Football League Quarterbacks: A Comparative Analysis of Performance Metrics

**DOI:** 10.7759/cureus.114758

**Published:** 2026-08-18

**Authors:** William J Gadbois, Hunter Cohn, Hadi Elmenini, Tavan Zadeh, Rahul Vaidya

**Affiliations:** 1 Medicine, Wayne State University School of Medicine, Detroit, USA; 2 Orthopedic Surgery, Detroit Medical Center, Detroit, USA

**Keywords:** athletic performance metrics, fantasy football metrics, nfl quarterbacks, passer rating, post-surgical recovery, quarterback injuries, return to performance, return to pre-injury performance, total qbr

## Abstract

Background

Return to play (RTP) is commonly used to assess recovery after injury but does not reflect post-injury performance quality. Return to pre-injury performance (RPIP) offers a more informative outcome, yet its application to National Football League (NFL) quarterbacks and the optimal performance metric remains unclear. This study compared commonly used quarterback performance metrics to determine whether any single measure effectively characterizes RPIP following injury requiring surgery.

Methods

This retrospective cohort study included NFL quarterbacks who had sustained a first injury requiring surgical intervention between 2006 and 2022, missed regular-season games, and had at least one full pre-injury season. Performance was assessed using Fantasy Football Points, ESPN Total Quarterback Rating (QBR), Passer Rating, and Passing Attempts. Pre-injury baseline was defined as each quarterback’s peak single-season performance within the three years preceding injury to provide a stable estimate of individual pre-injury capacity. RPIP was calculated as (peak post-injury performance / pre-injury baseline) × 100 and expressed as a percentage, with 100% indicating complete return to pre-injury peak performance. Paired t-tests compared pre- and post-injury performance, and one-way analysis of variance (ANOVA) assessed differences in recovery magnitude across metrics.

Results

Seventy-three quarterbacks met inclusion criteria. Mean RPIP was approximately 94% for Fantasy Points, 107% for QBR, 97% for Passer Rating, and 91% for Passing Attempts. No statistically significant differences were observed between pre- and post-injury peak performance for any metric, with only small effect sizes across all measures (Cohen's d range: −0.19 to 0.10), suggesting minimal practical differences between pre- and post-injury performance. No statistically significant differences in recovery magnitude were detected across the performance metrics, although formal equivalence was not established.

Conclusion

Common quarterback performance metrics demonstrate similar overall patterns of post-injury decline and recovery. While certain metrics may offer advantages within specific subgroups, no single measure demonstrated superior overall sensitivity for assessing RPIP. These conclusions are based on the absence of statistically significant between-metric differences rather than formal equivalence testing and should be interpreted accordingly.

## Introduction

Quarterbacks occupy a uniquely central role in American football, functioning as the tactical and emotional anchor of nearly every offensive play. They touch the ball on virtually every snap, direct the flow of the game, and often dictate a team’s overall success. Because of this centrality, injuries or declines in quarterback performance have disproportionate effects on both individual careers and team outcomes [1]. Evaluating not just whether a quarterback returns to play, but how well they return, is therefore essential to understanding true recovery quality and sustained competitive performance [2].

Traditionally, return to play (RTP) has been the standard measure of post-injury recovery [3]. While RTP indicates whether and when an athlete returns to competition, it does not capture the quality of their performance upon return [4,5]. A player may meet the criteria to rejoin competition but perform far below their pre-injury level. Return to pre-injury performance (RPIP), defined as the percentage of a player’s pre-injury output regained after recovery, offers a more meaningful, quantitative measure of recovery [6-8]. Recent studies have applied RPIP to professional athletes to better characterize recovery trajectories and to move beyond binary determinations of return [9-11]. However, despite growing recognition of RPIP’s value, its application to quarterbacks specifically has yet to be studied, and the optimal metric for assessing performance recovery in this position is unclear [1,5].

A range of statistics are used to evaluate quarterback performance, each emphasizing different aspects of play. The National Football League (NFL) introduced the passer rating system in 1973 to provide a standardized method for comparing quarterbacks across eras [12]. Passer rating continues to be the league’s official measure of quarterback performance and is calculated from four components: completion percentage, yards per attempt, touchdown percentage, and interception percentage. However, passer rating was designed in the game’s earlier era, when passing volume and offensive complexity were lower, and it omits important contextual factors such as sacks, rushing contributions, and situational impact.

To address these limitations, ESPN introduced Total Quarterback Rating (QBR) in 2011 in collaboration with statisticians, analysts, and former quarterbacks [9]. Using data from tens of thousands of NFL plays, QBR was developed to capture a more complete picture of quarterback performance by integrating play context, game situation, and both passing and rushing effectiveness [13]. Following its introduction, ESPN retrospectively applied QBR to the five preceding NFL seasons, enabling performance evaluation dating back to the 2006 season.

Passing attempts, by contrast, offer a straightforward indicator of volume: how often a quarterback is relied upon to throw the ball. Although this measure does not directly assess efficiency, it reflects offensive workload, coaching confidence, and player availability, making it a useful complementary measure of post-injury recovery. However, because passing attempts quantify opportunity rather than effectiveness, they do not capture whether performance at that volume remains efficient. 

More recently, fantasy football scores have been used as a composite, publicly available measure of player performance in sports medicine research [14-19]. These systems aggregate real-world statistics into a single interpretable value encompassing productivity (e.g., yardage and touchdowns), efficiency (e.g., completion percentage), and negative plays (e.g., interceptions and fumbles), while also capturing performance components such as quarterback rushing that are not fully reflected in traditional passing metrics. By weighing both positive and negative contributions across a season, fantasy scoring provides a multidimensional representation of offensive production. Whether this composite measure characterizes RPIP similarly to more established quarterback metrics has not been evaluated. 

Together, these four measures, Pass Attempts, Passer Rating, QBR, and Fantasy Score, capture distinct dimensions of quarterback performance, including workload, passing efficiency, contextual effectiveness, and overall production. Whether these measures characterize post-injury recovery similarly or provide different perspectives on RPIP remains unclear. The purpose of this study was to compare how these commonly used performance metrics characterize RPIP following injury requring surgery in NFL quarterbacks. We hypothesized that no single metric would demonstrate superior sensitivity to characterize RPIP. By examining similarities and differences in recovery patterns across metrics, this analysis aims to clarify how performance recovery is measured and to inform the selection of outcome measures for future research and clinical interpretation.

## Materials and methods

Study design and population

This is a retrospective cohort study of NFL quarterbacks who sustained an injury requiring surgery between 2006 and 2022, which coincided with the introduction and availability of ESPN’s total QBR metric. Each player’s first surgically treated injury was considered the index event. Any discrepancies in player eligibility or data abstraction were resolved through consensus discussion among the study investigators; because final inclusion decisions were adjudicated collaboratively, formal inter-reviewer reliability statistics were not assessed. Players were required to have at least one full pre-injury season to establish a performance baseline. Quarterbacks were excluded if they lacked sufficient pre- or post-injury data for comparison or if their injury did not result in missed regular-season games. Subsequent injuries requiring surgery were tracked to evaluate long-term durability and contextualize post-injury trends.

Data sources and verification

All data were obtained from publicly available sources. Injury information was collected from Spotrac and cross-referenced with Pro Football Reference, ESPN, and verified news reports to confirm accuracy. Cases with missing or ambiguous injury details were included only when the injury type, surgical intervention, and timing could be corroborated across multiple independent sources; otherwise, the case was excluded from analysis. Performance data, including Total QBR, Passer Rating, Passing Attempts, and Fantasy Football scores, were collected from ESPN [20], Pro Football Reference [21], and FantasyData [22]. Fantasy football scores were calculated using a single standardized scoring system obtained from FantasyData [22] and applied uniformly across all quarterbacks to ensure consistency and reproducibility. Data abstraction was conducted independently by four reviewers, and discrepancies were resolved through structured consensus review to ensure consistency and accuracy; because final values were established by consensus adjudication, formal inter-reviewer reliability statistics were not assessed.

Performance metrics

Four key indicators of quarterback performance were analyzed and are outlined in Table 1.

**Table 1:** Overview of quarterback evaluative metrics *The example fantasy scoring system presented in the table is illustrative and does not include all scoring components; the complete scoring system is available on the FantasyData website [22].

Metric​	Description​	Scoring System​
Total QBR ​[13]	ESPN’s proprietary composite efficiency metric (scale 0–100) incorporating play context, expected points added, and decision-making. ​	Weighted composite of Expected Points Added (EPA) per play, adjusted for situational context (down, distance, field position, score differential, and win probability). Plays are weighted by leverage and normalized on a 0–100 scale, where 50 = league-average performance. ​
Passer Rating ​[12]	The traditional NFL efficiency measure derived from completion percentage, yards per attempt, touchdowns, and interceptions. ​	Composite index based on four component rates—completion efficiency, yards per attempt, touchdown frequency, and interception avoidance—each scaled to reflect passing efficiency. The resulting average is normalized to a theoretical range of 0–158.3, where ~100 represents league-average quarterback play. ​
Passing Attempts ​	A measure of workload and offensive involvement, representing passing volume per season. ​	Aggregate count of all recorded pass attempts, including completions and incompletions; serves as a proxy for offensive opportunity and usage. ​
Fantasy Football Score ​[22]	A composite measure integrating productivity (yardage, touchdowns), efficiency, and negative plays (interceptions, fumbles) into a single interpretable value. ​	Summative point-based system assigning weighted values to offensive outcomes to produce a single composite score reflecting weekly or seasonal productivity. ​ Example scoring system*: +1 pt / 25 pass yards, +4 per pass touchdown, –2 per interception, +1 per 10 rush/rec yards, +6 per rush/rec touchdown, –2 per fumble lost, and +2 per two-point conversion. ​

Passing attempts and fantasy points were aggregated across each season to reflect cumulative workload and volume-based productivity, while passer rating and QBR were averaged across each season to preserve interpretability on their native scales. Because each metric was compared within players using the same measure before and after injury, analyses focused on within-metric changes rather than direct comparisons of raw values across metrics.

Recovery magnitude was assessed using RPIP, calculated in the primary analysis as the ratio of peak post-injury to peak pre-injury performance for each metric, and no additional normalization was performed. For each quarterback, the primary pre-injury baseline was defined as the best single-season performance in the three years preceding injury, while post-injury values included all seasons following the index surgery. This approach was selected because the study sought to determine whether quarterbacks were able to regain their highest demonstrated level of performance rather than their average level of play. However, because use of peak performance may overestimate baseline relative to a player’s typical level of play, a sensitivity analysis was performed using mean performance across all available seasons within the same three-year pre-injury window as the alternative baseline. RPIP and the proportion of quarterbacks achieving RPIP were recalculated for each metric using this mean baseline and compared descriptively with the primary peak-baseline analysis. The results of this sensitivity analysis are presented in the appendix.

Return to pre-injury performance (RPIP)

For each metric, RPIP was calculated as the ratio of the player’s peak post-injury season performance to their pre-injury baseline, expressed as a percentage. RPIP quantified the proportion of pre-injury ability regained after recovery.

Additional variables and subgroup analysis

Additional variables collected included: age at injury, seasons played, injury location, injury region (upper vs. lower extremity), injury laterality (right vs. left), hand dominance, body mass index (BMI), 40-yard dash time, and average rushing attempts per game. Players were also separated into quartiles of pre-injury performance within position-based metrics to identify high- versus low-performing subgroups. These variables were used for exploratory subgroup analysis.

Patient and public involvement

Patients and/or members of the public were not involved in the design, conduct, or reporting of this research. The study consisted of a retrospective analysis of publicly available, de-identified performance data, which precluded direct patient or public involvement.

Statistical analysis

Descriptive statistics were used to summarize demographic and performance data. Pre- and post-injury comparisons for each metric were conducted using paired t-tests, with Cohen’s d effect sizes calculated to quantify the standardized mean difference. To compare recovery across performance metrics, RPIP values, calculated as the ratio of peak post-injury to peak pre-injury performance for each quarterback, were compared across the four metrics using one-way analysis of variance (ANOVA) with post hoc Tukey testing. This analysis was exploratory and was intended to compare mean RPIP magnitudes across metrics rather than establish formal equivalence or superiority. Formal testing of the normality of RPIP distributions was not performed; therefore, the results of this parametric analysis should be interpreted cautiously, particularly because ratio measures may be influenced by skewness or extreme values. 

As a sensitivity analysis, RPIP was recalculated using mean performance across all available seasons within the three-year pre-injury window as the baseline. Mean-baseline RPIP values and the proportion of quarterbacks achieving RPIP were compared descriptively with the primary peak-baseline results. Paired t-tests were used for subgroup comparisons, with corresponding effect sizes calculated where appropriate. These analyses were intended to assess differences in observed recovery patterns across metrics rather than to establish formal equivalence between performance measures. Because multiple subgroup and categorical comparisons were performed, these analyses may carry an increased risk of Type I error and should be interpreted as exploratory. Statistical significance was defined as p<0.05.

## Results

Cohort characteristics

Between 2006 and 2022, a total of 87 NFL quarterbacks were identified who sustained a first injury requiring surgical intervention and met initial inclusion criteria. Fourteen quarterbacks were excluded due to insufficient pre-injury seasons to establish a reliable baseline, resulting in a final analytic cohort of 73 quarterbacks. This cohort formed the basis for all subsequent pre- versus post-injury comparisons across quarterback performance metrics.

Comparison of RPIP across quarterback metrics

**Figure 1:**
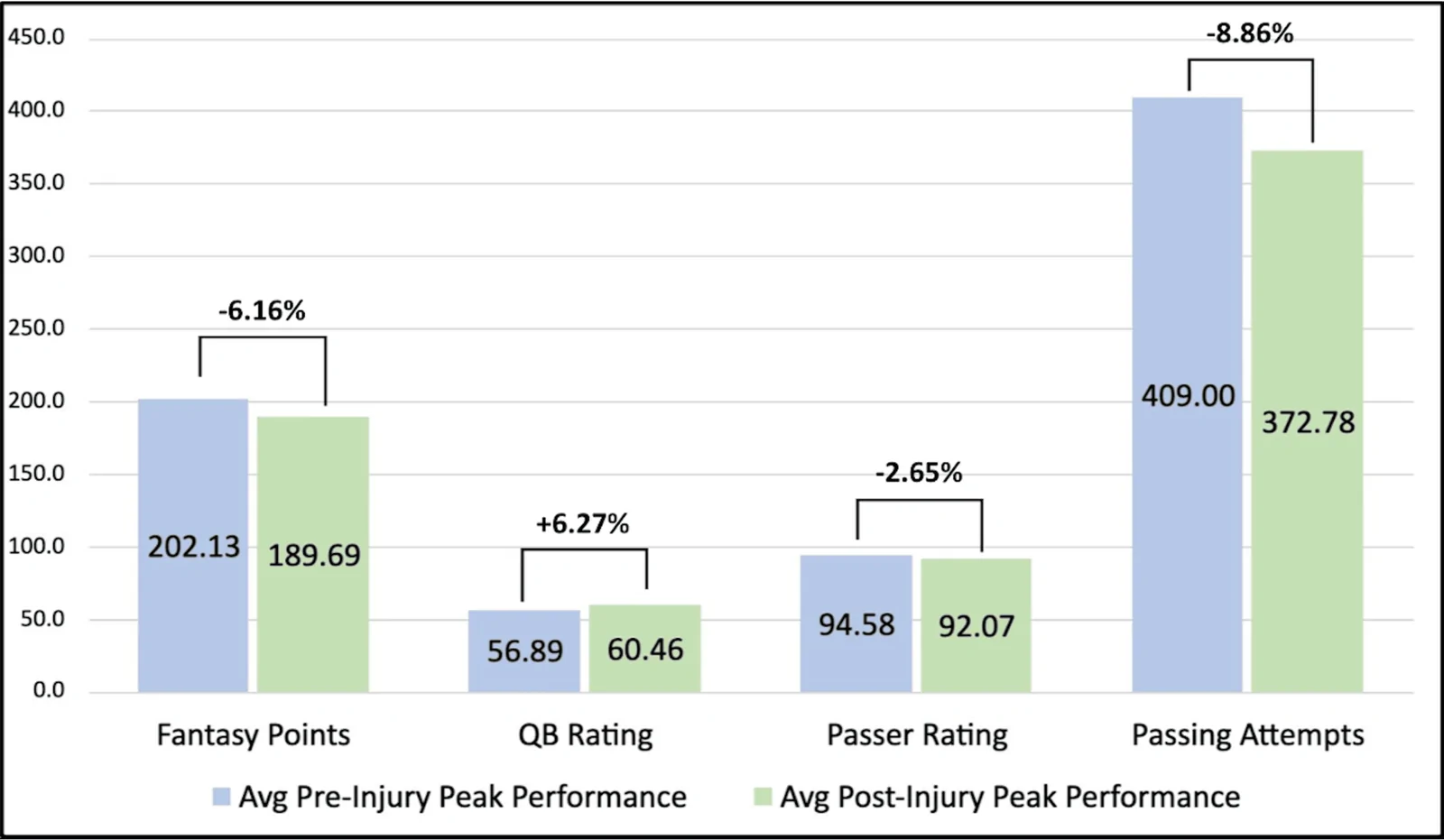
Return to pre-injury performance across quarterback metrics Comparison of average peak pre-injury and post-injury quarterback (QB) performance across four commonly used metrics: Fantasy Points, Total QBR, Passer Rating, and Passing Attempts. Bars represent mean peak values before and after injury, with percentage changes from pre-injury performance shown above each metric. These percentage changes correspond to return to pre-injury performance (RPIP), calculated as (peak post-injury performance / pre-injury baseline) × 100 and expressed as a percentage. Fantasy Points and Passing Attempts demonstrated the largest proportional declines, while Total QBR showed a modest post-injury increase.

Figure 1 illustrates average peak pre-injury and post-injury performance across four quarterback metrics: Fantasy Points, Total QBR, Passer Rating, and Passing Attempts.

Across metrics, post-injury performance generally declined relative to pre-injury peaks, although the magnitude and direction of change varied. Fantasy Points declined by 6.16%, Passer Rating by 2.65%, and Passing Attempts by 8.86%, while total QBR showed a modest post-injury increase of 6.27%. When framed in terms of RPIP, these changes correspond to RPIP values of approximately 94% for Fantasy Points, 107% for QBR, 97% for Passer Rating, and 91% for Passing Attempts. No single metric demonstrated significantly greater recovery than the others, with RPIP values ranging from approximately 91% to 107% across the four measures.

Table 2 summarizes the statistical comparisons of pre- and post-injury peak performance across the four quarterback metrics.

**Table 2:** Statistical analysis of pre- and post-injury performance across quarterback metrics

Quarterback metric​	Pre-Injury Peak​	Post-Injury Peak​	T-Test​ & Cohen’s d	ANOVA Tukey test results​			
				Vs Fantasy​	Vs QBR​	Vs PR​	Vs PA​
Fantasy Points​	202.13​	189.69​	t=0.60​ p=0.55 d=​-0.11	--​	Q=1.21​ p=0.83​	Q=0.75​ p=0.95​	Q=1.80​ p=0.58​
QBR​	56.89​	60.46​	t=-0.82 ​ p=0.41 d=0.10​	Q=1.21​ p=0.83​	--​	Q=0.46​ p=0.99​	Q=3.01​ p=0.15​
Passer Rating (PR)​	94.58​	92.07​	t=0.59​ p=0.56​ d=​-0.08	Q=0.75​ p=0.95​	Q=0.46​ p=0.99​	--​	Q=2.55​ p=0.27​
Passing Attempts (PA)​	409.00​	372.78​	t=1.04​ p=0.30​ d=​-0.19	Q=1.80​ p=0.58​	Q=3.01​ p=0.15​	Q=2.55​ p=0.27​	--​

Paired t-tests demonstrated no statistically significant differences between pre- and post-injury peak values for Fantasy Points, Total QBR, Passer Rating, or Passing Attempts. Cohen's d was calculated using the paired difference (pre-injury minus post-injury); therefore, negative values indicate lower post-injury performance relative to baseline, whereas positive values indicate higher post-injury performance. Corresponding Cohen’s d values were uniformly small in magnitude (range: −0.19 to 0.10), indicating minimal practical differences between pre- and post-injury performance across all four measures. To directly compare the magnitude of change captured by each metric, an ANOVA with post-hoc Tukey testing was performed and similarly revealed no significant differences in mean pre- to post-injury change between the four measures.

Collectively, these findings indicate that no statistically significant differences in recovery magnitude were detected between the four metrics, with no single measure demonstrating greater sensitivity to post-injury performance change within the scope of these analyses.

Subgroup analyses were performed to assess whether pre- to post-injury changes differed across clinically and demographically relevant strata, including BMI, injury location (upper vs lower extremity), career duration, and injury laterality relative to hand dominance.

Paired t-tests comparing pre- and post-injury peak performance within each subgroup are summarized in Table 3 for all four quarterback metrics (Fantasy Points, Total QBR, Passer Rating, and Passing Attempts).

**Table 3:** Sub-group analysis of pre- and post-injury performance across quarterback metrics *Values indicate statistically significant differences (p<0.05).​

Subgroup​	Criteria​	Sample size ​ (# of players)​	t-test results and Cohen’s d for change in pre-injury to post-injury performance​			
			Fantasy Points​	QBR​	Passer Rating​	Pass Attempts​
BMI​	BMI <28​	38​	p<0.01*​ d=​0.19	p=0.98​ d=​-0.08	p=0.81 d=​0.06​	p=0.20​ d=​0.30
	BMI >28​	35​				
Injury Location​	Upper Extremity​	36​	p=0.84​ d=​-0.10	p=0.46 d=​-0.06​	p=0.87​ d=​-0.06	p=0.69​ d=​0.06
	Lower extremity​	37​				
Seasons Played​	<10 seasons​	35​	p=0.10​ d=​0.39	p=0.37 d=​0.54​	p=0.10 d=​0.39​	p=0.06​ d=​0.45
	>10 seasons​	38​				
Injury to Dominant Extremity​	Yes​	35​	p=0.11​ d=​-0.38	p=0.66 d=​0.09​	p=0.65 d=​0.10​	p<0.05*​ d=​-0.46
	No​	38​				

Overall, subgroup effects were limited and inconsistent across metrics, with statistically significant differences observed only in isolated comparisons. Most subgroup Cohen’s d values were small in magnitude, although select comparisons, such as career duration effects on QBR (d=0.54) and passing attempts (d=0.45), approached moderate effect sizes.

Notably, quarterbacks sustaining injuries to the non-dominant side demonstrated lower passing attempts compared to those with dominant-side injuries, while quarterbacks with BMI <28 exhibited lower fantasy football scores relative to quarterbacks with BMI ≥28.

Given the modest sample sizes within subgroup strata and the number of comparisons performed, these findings should be interpreted as exploratory and hypothesis-generating rather than confirmatory. No subgroup demonstrated uniform or consistent effects across all performance measures, suggesting that demographic and injury-related factors exert variable and metric-specific influences on observed return to pre-injury performance.

## Discussion

Overview of key findings

In this study, we compared four commonly used quarterback performance metrics, Fantasy Points, ESPN Total QBR, Passer Rating, and Passing Attempts, to evaluate RPIP following injury requiring surgery. The principal finding is that all four metrics demonstrated similar patterns of post-injury decline and partial recovery, with no single measure emerging as clearly superior in detecting changes from pre- to post-injury peak performance. Across the cohort, paired comparisons and between-metric analyses showed similar magnitudes of RPIP, supporting the conclusion that these metrics broadly track recovery in parallel rather than capturing fundamentally distinct recovery trajectories. Importantly, the lack of observed differences should not be interpreted as evidence of equivalent sensitivity or validity across metrics, but rather as an absence of a clearly superior performer within the scope of the present analyses.

Interpretation and implications

Taken together, these findings suggest that quarterbacks, as a position group, demonstrate substantial resilience following injury, frequently returning to a large proportion of their pre-injury performance. While absolute recovery was often incomplete, the observed RPIP across metrics indicates that many quarterbacks are able to re-establish effective, high-level play after surgery. This aligns with the unique role of the quarterback, where performance depends not only on physical capacity but also on decision-making, experience, and schematic integration - factors that may facilitate functional recovery even when physical attributes are altered [2]. Age, accumulated experience, and prior surgical burden may also influence how different performance metrics reflect RPIP following injury [23]. For example, older or more experienced quarterbacks may preserve efficiency-based measures such as QBR or passer rating through decision-making, anticipation, and schematic familiarity, even when workload-based measures such as passing attempts or fantasy production decline. Similarly, quarterbacks with multiple prior surgeries or greater cumulative musculoskeletal burden may demonstrate metric-specific recovery patterns depending on whether performance is driven more by efficiency, mobility, or volume. From an RPIP perspective, these results reinforce the importance of evaluating recovery quality rather than relying solely on return-to-play milestones.

Although no single metric demonstrated superior sensitivity to post-injury change in this cohort, the similar recovery trends observed across measures have important practical implications. In particular, these findings suggest that simpler, more transparent measures may offer similar descriptive utility in certain return-to-performance contexts, even though equivalent sensitivity or construct validity cannot be assumed. Fantasy football scores, while originally designed for recreational use, represent one such measure that is publicly available, easily calculated, widely understood by clinicians, analysts, players, and fans, and integrates multiple dimensions of performance, including volume, efficiency, and negative plays, into a single interpretable value. When fantasy scores demonstrated recovery trends similar to QBR and passer rating in this cohort, their accessibility and reproducibility may make them a useful option for sports medicine and performance research when more complex metrics are unavailable. 

Subgroup analyses revealed limited and non-uniform differences across demographic and injury-related strata, though a small number of statistically significant findings warrant consideration. Specifically, quarterbacks with lower BMI demonstrated lower fantasy football production following injury, and quarterbacks sustaining injuries to the non-dominant side exhibited reduced passing attempts compared to those with dominant-side injuries. These isolated findings may reflect how certain metrics are differentially influenced by variables such as player build, biomechanics, or role within offensive schemes [24,25]. For example, fantasy scoring, by incorporating rushing output and cumulative production, may be more sensitive to changes in mobility or durability that disproportionately affect lighter quarterbacks. Similarly, reductions in passing volume may be more apparent when injury laterality alters throwing mechanics, practice efficiency, or coaching trust. However, given the absence of consistent effects across metrics and subgroups, these results should be interpreted as hypothesis-generating rather than definitive.

Overall, this analysis supports a reframing of quarterback recovery assessment: rather than searching for a single “best” performance metric, emphasis should be placed on selecting measures that are appropriate, interpretable, and fit for purpose. When multiple metrics convey similar recovery patterns, practical considerations such as transparency, accessibility, and multidimensional representation may help guide metric selection, while recognizing that further validation is needed to establish comparative validity and sensitivity. 

Comparison to existing literature

Our findings both validate and extend prior work on post-injury quarterback performance. Across multiple commonly used metrics, quarterbacks demonstrated relatively preserved RPIP, reinforcing the idea - suggested in earlier injury-specific studies - that quarterbacks may recover competitive effectiveness more reliably than many other positions. Importantly, most existing analyses have focused on discrete injuries [23,26]. By examining injuries requiring surgery more broadly rather than restricting the sample to one diagnosis, this study suggests that the “quarterback resilience” signal is not limited to a single injury type and may reflect position-level features, including decision-making, experience, schematic control, and role stability, that buffer performance outcomes after physical setbacks.

This work also adds to the growing body of literature evaluating fantasy points as a performance outcome measure. Prior studies have used fantasy scoring to quantify post-injury change in specific sports and injuries, generally showing measurable declines and incomplete return in many cohorts [14-19]. Here, fantasy points demonstrated recovery patterns similar to those observed with established quarterback metrics (passer rating, Total QBR, and passing volume), with no statistically significant differences detected in overall recovery magnitude across the measures evaluated. The observed comparability across metrics is noteworthy, as it indicates that a transparent and publicly available composite measure demonstrated recovery trends comparable to those observed with more complex or proprietary alternatives in this cohort, without establishing formal equivalence in sensitivity or validity. This finding suggests that fantasy points may serve as a practical outcome measure for return-to-performance research, particularly in contexts where reproducibility, transparency, and accessibility are important, although additional validation is needed before broader adoption.

Limitations and future directions

This study has several important limitations. First, the study period was constrained by the introduction of ESPN Total QBR in 2006, which defined the earliest feasible start date for analysis. As QBR is a proprietary metric, its calculation could not be reconstructed or retrospectively applied to earlier seasons, limiting sample size and precluding longer longitudinal assessment using this measure. Although this restriction affected only one of the four evaluated metrics, it reduced the overall cohort available for cross-metric comparisons. Additionally, injury and surgical histories prior to NFL entry, including those sustained during collegiate play, were not consistently available and therefore not captured, raising the possibility that some quarterbacks had prior procedures that influenced baseline performance or recovery trajectories. Age at the time of injury may also have influenced both baseline performance and post-injury recovery, as changes in athletic performance associated with normal career progression could not be fully accounted for in this retrospective analysis. Additionally, restricting inclusion to quarterbacks who missed regular-season games may bias the cohort toward more severe injuries, as players who returned despite injury or underwent surgery without documented missed game time would not have been captured. Measures of injury severity, surgical technique, graft choice, and rehabilitation protocols were also not uniformly reported in publicly available sources and therefore could not be incorporated; injuries were classified broadly based on whether they required operative intervention.

Second, performance was assessed using peak pre- and post-injury seasons rather than continuous season-by-season trajectories. While this approach aligns with the study’s focus on RPIP and each athlete’s maximal demonstrated capacity, it may overestimate baseline performance and introduce upward bias in RPIP estimates relative to a player's typical level of play at the time of injury. More importantly, reliance on peak seasons reduces sensitivity to longitudinal recovery trajectories and may obscure shorter-term fluctuations, delayed recovery, transient declines, or context-dependent performance variability related to role changes, team transitions, or subsequent injuries. Additionally, volume-based metrics (Fantasy Points and Passing Attempts) were evaluated using cumulative seasonal totals, whereas rate-based metrics (QBR and Passer Rating) were evaluated using season-average values. Consequently, quarterbacks who played fewer games following injury could demonstrate lower cumulative production despite maintaining comparable per-game performance, potentially contributing to greater apparent declines in volume-based metrics relative to efficiency-based measures. Future studies using per-game normalization or other exposure-adjusted approaches may help clarify whether these differences reflect true recovery patterns or differences in playing opportunity. 

Third, although multiple clinically relevant subgroup analyses were performed, several findings were limited by modest sample sizes. Because multiple subgroup comparisons were conducted without adjustment for multiple testing, some statistically significant findings may represent chance associations (Type I error) rather than true subgroup effects. Accordingly, these subgroup findings should be interpreted as exploratory and hypothesis-generating rather than confirmatory.

The absence of consistent subgroup effects across metrics further suggests that larger cohorts may be necessary to clarify whether certain player characteristics meaningfully modify return-to-performance trajectories. Additionally, RPIP was calculated as a ratio and may not have been normally distributed. Because formal distributional diagnostics were not performed, the ANOVA findings may have been influenced by skewness or extreme observations and should also be considered exploratory. Future analyses should evaluate these assumptions and confirm the findings using nonparametric methods or bootstrapped confidence intervals. 

Future research should extend this framework to larger datasets as additional seasons of QBR data become available and should consider applying similar comparative approaches to other quarterback-specific metrics, including advanced non-proprietary efficiency measures. Expanding this methodology to other positions and professional leagues may further clarify whether composite performance metrics such as fantasy scoring provide generalizable advantages for evaluating recovery. Finally, integrating clinical variables such as injury severity, surgical technique, and rehabilitation timelines could improve predictive modeling of RPIP and refine outcome assessment in sports medicine research.

## Conclusions

Quarterbacks occupy a uniquely central role in team performance, and injuries at this position can have cascading effects on both individual careers and organizational success. Accurately evaluating RPIP, rather than simply return to play, is therefore essential for understanding recovery in this population. In this analysis, commonly used quarterback performance metrics demonstrated similar patterns of post-injury decline and recovery, and no statistically significant differences were detected in the magnitude of recovery across the measures evaluated. Consequently, no single metric emerged as clearly superior for assessing RPIP within the scope of this study, although formal equivalence in validity or sensitivity was not established. These findings reinforce the resilience of quarterbacks as a position while highlighting that multiple commonly used performance metrics may provide comparable perspectives on recovery. Fantasy football-based performance measures represent one transparent, accessible, and reproducible option among these metrics when more complex or proprietary measures are unavailable. However, this recommendation is preliminary and requires validation across additional player positions, sports, and clinical outcome benchmarks before widespread adoption. Overall, these findings support selecting performance metrics based on study objectives, interpretability, and data availability while contributing to a more standardized approach to evaluating quarterback recovery following significant injury.

## Appendices

**Table 4:** Sensitivity analysis of return to pre-injury performance using peak versus mean pre-injury baselines RPIP was calculated using either peak single-season performance within the three-year pre-injury period (primary analysis) or mean performance across available seasons within the same period (sensitivity analysis). Use of mean baseline resulted in higher RPIP estimates and a greater proportion of quarterbacks achieving RPIP across all four metrics, while the overall pattern across measures remained broadly consistent. The systematically higher estimates with mean baseline reflect its lower performance threshold and frequently resulted in RPIP values exceeding 100%, indicating recovery beyond a quarterback’s average pre-injury performance without necessarily representing return to their highest demonstrated level. These findings support the use of peak performance as the primary baseline for the study’s intended construct of return to pre-injury performance, while demonstrating that the overall comparative interpretation across metrics was not dependent on this baseline definition. RPIP, return to pre-injury performance; QBR, Total Quarterback Rating.

	Fantasy Score		QBR		Passer Rating		Passing Attempts	
Outcome	Peak Baseline	Mean Baseline	Peak Baseline	Mean Baseline	Peak Baseline	Mean Baseline	Peak Baseline	Mean Baseline
Pre-injury baseline performance	202.13	136.94	56.89	42.50	94.58	79.24	409.00	291.85
Post injury performance as % of baseline	93.84%	138.52%	106.27%	142.27%	97.35%	116.20%	91.14%	127.73%
Achieved RPIP, n (%)	28 (38%)	46 (63%)	37 (51%)	55 (75%)	42 (57%)	59 (81%)	28 (38%)	48 (66%)
